# Cardioprotective Effect of Cilostazol on Ischemia-Reperfusion Injury Model

**DOI:** 10.21470/1678-9741-2020-0651

**Published:** 2022

**Authors:** Mazlum Sahin, Corc Baytaroglu, Emrah Sevgili

**Affiliations:** 1 Department of Cardiovascular Surgery, Avcilar Hospital, Istanbul, Turkey.; 2 Department of Cardiology, Avcilar Hospital, Istanbul, Turkey.

**Keywords:** Adenosine Triphosphate, Cilostazol, Inflammation, Myocardial Ischemia, Superoxide Dismutase

## Abstract

**Introduction:**

To clarify the potential protective role of cilostazol on rat myocardial cells with ischemia-reperfusion injury (IRI) models.

**Methods:**

The study was conducted with three groups of 10 Wistar rats (control group, rats without any coronary ischemia; sham group, rats with coronary ischemia but without cilostazol administration; and cilostazol group, rats with coronary ischemia and cilostazol administration). The level of myocardial injuries was measured by analyzing cardiac troponin T and creatine kinase MB levels in blood samples. In tissue samples, adenosine triphosphate (ATP), nitric oxide, superoxide dismutase (SOD), and malondialdehyde were used to determine the amount of tissue damage. Tissues were stained with hematoxylin-eosin method, and samples were examined under light microscope.

**Results:**

The mean level of ATP was 104.4 in the cilostazol group and 149.1 in the sham group (*P*=0.044). SOD level was significantly higher in the cilostazol group than in the sham group (2075.3 *vs*. 1783.7, *P*=0.043). According to histopathological examination, all samples were classified as G0 in the control group. In the sham group, one sample was categorized as G1, six samples as G2, and three samples as G3. In the cilostazol group, nine samples and one sample were categorized as G1 and G2, respectively (*P*=0.011).

**Conclusion:**

Cilostazol has beneficial effects on Wistar rats’ myocardial cells in regard to decreasing inflammatory process, necrosis, and fibrosis. Our findings revealed that the use of cilostazol significantly decreased ATP and increased SOD levels in Wistar rats’ myocardial cells after IRI.

**Table:** Abbreviations, acronyms & symbols

ATP	= Adenosine triphosphate
CK-MB	= Creatine kinase MB
cTnT	= Cardiac troponin T
IRI	= Ischemia-reperfusion injury
LAD	= Left anterior descending
MDA	= Malondialdehyde
NO	= Nitric oxide
PKA	= Protein kinase A
SOD	= Superoxide dismutase

## INTRODUCTION

Ischemia-reperfusion injury (IRI) is defined as a tissue injury due to return of blood supply after a period of blood flow insufficiency associate with hypoxia or anoxia^[1]^. The preferred treatment modality for acute myocardial infarction is reperfusion of infarct-related myocardial cells as early as possible. However, this process risks intracellular calcium overload, oxidative stress, and mitochondrial permeability transition pore opening^[2]^. Previous studies have alternatively suggested therapeutic hypothermia, therapeutic hyperoxemia, mesenchymal stem cell therapy, and various medical treatments including cyclosporine, metformin, and cannabinoids^[3]^.

Cilostazol is a quinolone derivative with antithrombotic, antiplatelet, vasodilatory, and antimitogenic effects. Put simply, cilostazol reversibly inhibits phosphodiesterase 3A enzyme, which increases the cyclic adenosine monophosphate and protein kinase A (PKA) levels. The PKA immediately decreases platelet aggregation and smoothes muscle cell contractions^[4]^. Previous reports focused on the protective role of cilostazol on IRI have controversial results. Santos et al., investigating the protective effect of cilostazol on isolated rabbit femoral arteries, claimed that cilostazol has improved vascular reactivity after revascularization^[5]^. However, Neto et al. found no beneficial effect of cilostazol on kidney and skeletal striated muscle in rats with IRI models^[6]^. In contrast, O’Donnell et al. found positive effects of cilostazol on exercise-induced IRI in 90 patients with peripheral artery disease^[7]^.

Previous studies have examined the preventative effect of cilostazol on peripheral arteries with IRI models, but none investigated the beneficial effect of cilostazol on myocardial cells. In the present study, we aim to clarify the potential protective role of cilostazol on rat myocardial cells with IRI models.

## METHODS

The present study was approved by the Vakif Gureba University Ethics Committee, number 2017/115. The study was performed at the Bezmialem Vakif University and involved a total of 30 10-month-old Wistar rats, with an average weight of 300 grams. The Wistar rats were divided into three groups of 10: the control group, the sham group, and the cilostazol group.

- Control group (n=10): Wistar rats without any coronary ischemia.
- Sham group (n=10): Wistar rats with coronary ischemia but without cilostazol administration.
- Cilostazol group (n=10): Wistar rats with coronary ischemia and cilostazol administration.

### Surgical Technique

After applying ketamine anesthesia to the rats, the heart was revealed with an incision from the left third intercostal space, and the left anterior descending (LAD) coronary artery was determined. The proximal part of LAD was ligated with propylene 7.0. The relevance of LAD ligature was confirmed by the absence of pulse and flow by intraoperative Dopplermetry. The solution of cilostazol was given intraperitoneally as a 20 mg/kg bolus five minutes after coronary ischemia. After the 30-minute ischemia period, ligation was removed, and ischemia was terminated (reperfusion period). Reperfusion was performed for 120 minutes, after which the rats were sacrificed by cervical dislocation. After opening the thorax, intra-aortic blood was removed, and the hearts were excised.

The level of myocardial injuries was measured by analyzing cardiac troponin T (cTnT) and creatine kinase MB (CK-MB) levels in blood samples. The amount of damage in tissue samples was determined by measuring adenosine triphosphate (ATP), nitric oxide (NO), superoxide dismutase (SOD), and malondialdehyde (MDA) levels.

Cardiac tissue was fixed in 10% buffered formalin for one week. After fixation, Wistar rats’ hearts were dissected into 3-mm thick horizontal sections containing both ventricles. Tissues were stained using the hematoxylin-eosin method, and samples were examined under a light microscope. If IRI was noted, the following grading system was used.

- G0: No abnormal changes.
- G1: Swelling in myocardial cells (cloudy swelling, hydropic degeneration), necrosis in a small number of sparsely distributed cells.
- G2: Myofibrillar irregularity, focal necrosis.
- G3: Structural irregularity, extensive myocardial necrosis, edema, and inflammatory cell infiltration in the myocardium.

### Statistical Analysis

The IBM Corp. Released 2011, IBM SPSS Statistics for Windows, version 20.0, Armonk, NY: IBM Corp. software was used for statistical analysis. Histological analysis and expression of immunohistochemical markers were tested using nonparametric Mann-Whitney U test. Statistical significance was considered when two-tailed P-value < 0.05.

## RESULTS

In all Wistar rats, the experiment was completed in accordance with the protocol. The mean level of ATP was 104.4 in the cilostazol group and 149.1 in the sham group (*P*=0.044). In contrast, SOD level was significantly higher in the cilostazol group than in the sham group (2075.3 *vs*. 1783.7, *P*=0.043). MDA, NO, CK-MB, and cTnT levels were similar between the cilostazol group and the sham group (*P*=0.289, *P*=0.423, *P*=0.128, and *P*=0.370, respectively) (Table 1).

**Table 1:** Comparison of biochemical markers between the cilostazol and sham groups.

	Cilostazol	Sham	*P*-value
ATP*	104.4±33.7 (96.0)	149.1±55.7 (140.5)	0.044
SOD*	2075.3±318.7 (2122.9)	1783.7±280.1 (1740.7)	0.043
MDA*	42.0±11.1 (39.8)	37.7±5.9 (36.2)	0.289
NO*	445.6±63.5 (452.5)	463.2±24.6 (461.5)	0.423
CK-MB*	10.4±1.4 (9.9)	11.4±1.5 (11.2)	0.128
Troponin T*	52.7±21.9 (43.8)	59.9±11.5 (57.1)	0.370

* mean ± standard deviation (median value) ATP=adenosine triphosphate; CK-MB=creatine kinase MB; MDA=malondialdehyde; NO=nitric oxide; SOD=superoxide dismutase

A comparison between the cilostazol group and the control group showed no difference in ATP and SOD levels (*P*=0.923 and *P*=0.478, respectively). The levels of MDA and NO were higher in the cilostazol group than in the control group, but the differences were not significant (42.0 *vs*. 36.9, *P*=0.224 and 445.6 *vs*. 426.4, *P*=0.491, respectively). Moreover CK-MB and cTnT levels were comparable between the cilostazol group and the control group (*P*=0.146 and *P*=0.627) (Table 2).

**Table 2:** Comparison of biochemical markers between the cilostazol and control groups.

	Cilostazol	Control	*P*-value
ATP*	104.4±33.7 (96.0)	105.9±34.7 (116.5)	0.923
SOD*	1783.7±280.1 (1740.7)	1892.0±379.8 (1994.3)	0.478
MDA*	42.0±11.1 (39.8)	36.9±6.1 (34.8)	0.224
NO*	445.6±63.5 (452.5)	426.4±58.2 (443.5)	0.491
CK-MB*	10.4±1.4 (9.9)	11.3±1.4 (11.2)	0.146
Troponin T*	52.7±21.9 (43.8)	57.1±17.3 (55.1)	0.627

* mean ± standard deviation (median value) ATP=adenosine triphosphate; CK-MB=creatine kinase MB; MDA=malondialdehyde; NO=nitric oxide; SOD=superoxide dismutase

In the histopathological examination, all samples were classified as G0 in the control group. In the sham group, one sample was categorized as G1, six samples as G2, and three as G3. In the cilostazol group, nine samples were categorized as G1, and one as G2 (*P*=0.011) (Table 3).

**Table 3:** Comparison of pathology results between the groups.

	G0	G1	G2	G3	*P*-value
Control	10	0	0	0	0.011
Sham	0	1	6	3	
Cilostazol	0	9	1	0	

## DISCUSSION

Cilostazol’s utility has been proved for intermittent claudication in peripheral vascular pathologies in animal and case control human studies. Many studies have also suggested its beneficial effect on stroke^[8]^. Animal studies allow assessment of the impacts of cilostazol on myocardial cells with IRI; the present study revealed that it can decrease the inflammatory cascade, myocardial cell deterioration, and fibrosis.

The myocytes produce the energy needed for their vital functions under aerobic status, via fatty acid oxidation, glucose oxidation, and glycolysis. Insufficient oxygenation of myocardial cell leads to the exchange of anaerobic glycolysis for energy production, which decreases ATP levels by 65% at 15 minutes and by 90% at 40 minutes following ischemia^[9]^. However, blood supply and adequate oxygenation are critical for myocytes, as revealed by Jennings et al.^[10]^, who described myocardial IRI in canine hearts as a coronary ligation model. The authors proposed that reperfusion of myocardial cells promotes the development of necrosis^[10]^.

**Fig. 1:**
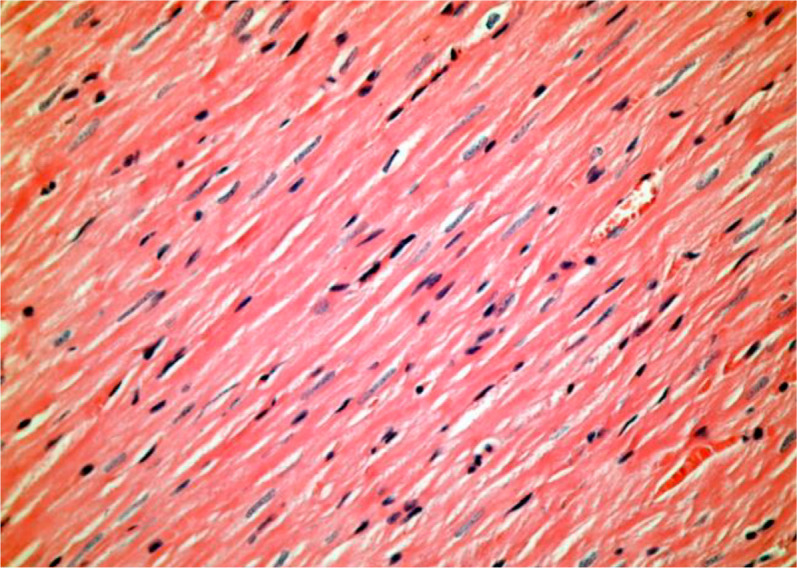
In the cilostazol group, natural myocardial tissue is observed (hematoxylin-eosin × 400).

**Fig. 2:**
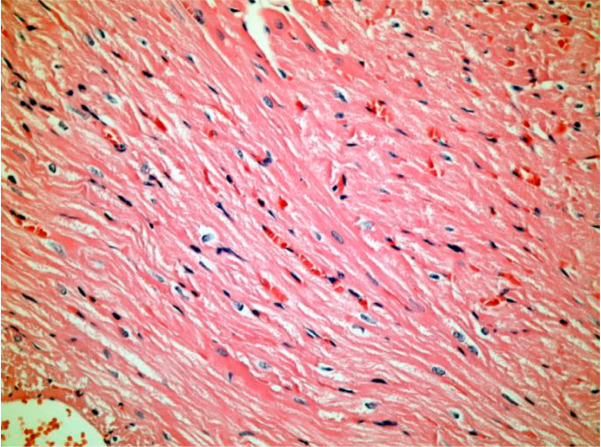
Hyperemia, edema between muscle fibers in the sham group (hematoxylin-eosin × 200).

Previous reports emphasized the importance of histopathological tissue alteration in determining IRI. Abel et al. proposed the four-level model, from G0 (no abnormal changes) to G3 (severe injury) to be used in the evaluation of IRI in myocardial cells^[11]^. Chen et al.^[12]^ used this classification to show the efficiency of diltiazem plus SOD on IRI^[12]^. In another study, Okada et al. investigated the protective effect of atenolol on myocardial cells in regard to the histopathological classification abovementioned^[13]^. In the present study, we found significantly less histopathological damage in Wistar rats’ myocytes when cilostazol was used (Figures 1 and 2).

The obstruction of blood flow is associated with hypoxia and deterioration of electron transport chain in mitochondria. Anaerobic status of cells influences ATP production and the amount of antioxidative agents, which may result in the deterioration of the ion-exchange channel function, enzyme activity in cytoplasm, and electrolyte imbalance. After reperfusion, blood flow with adequate oxygen level led to increased levels of reactive oxygen radicals, in contrast to lower levels of antioxidative agents^[14]^. Kristiansen et al.^[15]^ found higher ATP levels in IRI model in Sprague Dawley rats and concluded that reduction in ATP levels has beneficial effect on cell viability^[15]^. In the present study, we achieved significantly lower ATP levels in Wistar rats treated with cilostazol (*P*=0.044).

SODs are group of metalloenzymes that convert superoxide radicals to molecular oxygen and hydrogen peroxide. SODs are located in cytoplasm, nucleus, mitochondria, and the extracellular part of cell membrane; previous reports demonstrated the benefits of SODs in cancer prevention, regulation of inflammatory events, aging, neurodegenerative disease, and IRI models^[16]^. Ghio et al.^[17]^ investigated the possible effect of SODs on lung injury after air pollution in mice. The authors found that overexpression of extracellular SODs was associated with reduction in lung injury after exposure to oil fly ash^[17]^. In another study, Massini et al.^[18]^ showed that M40403 molecule (synthetic SOD molecule) had a protective effect on myocardial cells in IRI models^[18]^ In the present study, we found significantly higher SODs level in the cilostazol group.

### Limitations

The study has some limitations. Firstly, the present research is an animal experimental study, and as in all animal studies, it is unclear whether outcomes can be generalized to human clinical studies. Secondly, we evaluated six markers and myocardial histopathology, which are approved to use in IRI models in animal research; however, it should be noted that many other molecules, enzymes, and histological evaluation scales are available.

## CONCLUSION

In conclusion, cilostazol has beneficial effect on Wistar rats’ myocardial cells in regard to decreasing inflammatory process, necrosis, and fibrosis after 30-minute ischemia period and 120-minute reperfusion period. Moreover, our findings revealed that the use of cilostazol significantly decreased ATP and increased SOD levels in Wistar rats’ myocardial cells after IRI. Further research involving a greater number of subjects should be conducted to confirm these results.
